# CD47 cross-dressing by extracellular vesicles expressing CD47 inhibits phagocytosis without transmitting cell death signals

**DOI:** 10.7554/eLife.73677

**Published:** 2022-12-01

**Authors:** Yang Li, Yan Wu, Elena A Federzoni, Xiaodan Wang, Andre Dharmawan, Xiaoyi Hu, Hui Wang, Robert J Hawley, Sean Stevens, Megan Sykes, Yong-Guang Yang

**Affiliations:** 1 Key Laboratory of Organ Regeneration and Transplantation of the Ministry of Education, The First Hospital, and Institute of Immunology, Jilin University Changchun China; 2 Columbia Center for Translational Immunology, Columbia University Medical Center New York United States; 3 Lung Biotechnology PBC Silver Spring United States; 4 International Center of Future Science, Jilin University Changchun China; The Hebrew University of Jerusalem Israel; Indian Institute of Science Education and Research (IISER) India

**Keywords:** CD47, macrophages, extracellular vesicles, exosomes, xenotransplantation, tumor cells, None

## Abstract

Transgenic CD47 overexpression is an encouraging approach to ameliorating xenograft rejection and alloresponses to pluripotent stem cells, and the efficacy correlates with the level of CD47 expression. However, CD47, upon ligation, also transmits signals leading to cell dysfunction or death, raising a concern that overexpressing CD47 could be harmful. Here, we unveiled an alternative source of cell surface CD47. We showed that extracellular vesicles, including exosomes, released from normal or tumor cells overexpressing CD47 (transgenic or native) can induce efficient CD47 cross-dressing on pig or human cells. Like the autogenous CD47, CD47 cross-dressed on cell surfaces is capable of interacting with SIRPα to inhibit phagocytosis. However, ligation of the autogenous, but not cross-dressed, CD47 induced cell death. Thus, CD47 cross-dressing provides an alternative source of cell surface CD47 that may elicit its anti-phagocytic function without transmitting harmful signals to the cells. CD47 cross-dressing also suggests a previously unidentified mechanism for tumor-induced immunosuppression. Our findings should help to further optimize the CD47 transgenic approach that may improve outcomes by minimizing the harmful effects of CD47 overexpression.

## Introduction

CD47 is ubiquitously expressed and acts as a ligand of signaling regulatory protein (SIRP)α, a critical inhibitory receptor on macrophages and dendritic cells (DCs). Emerging evidence indicates that the CD47-SIRPα signaling pathway plays an important role in regulation of macrophage and DC activation, offering a promising intervention target for immunological disorders. CD47KO cells are vigorously rejected by macrophages after infusion into syngeneic wild-type (WT) mice, demonstrating that CD47 provides a ‘don’t eat me’ signal to macrophages (Oldenborg et al., 2000; Wang et al., 2007a). Xenotransplantation using pigs as the transplant source has the potential to resolve the severe shortage of human organ donors, a major limiting factor in clinical transplantation (Yang and Sykes, 2007). We reported that the strong rejection of xenogeneic cells by macrophages (Abe et al., 2002) is largely caused by the lack of functional interaction between donor CD47 and recipient SIRPα (Wang et al., 2007b, Ide et al., 2007; Navarro-Alvarez and Yang, 2014). These findings led to the development of human CD47 transgenic pigs that have achieved encouraging results in pig-to-nonhuman primate xenotransplantation (Tena et al., 2017; Nomura et al., 2020; Watanabe et al., 2020). In addition to macrophages, a sub-population of DCs also expresses SIRPα (Wang et al., 2007a, Guilliams et al., 2016). Importantly, CD47-SIRPα signaling also inhibits DC activation and their ability to prime T cells, and plays an important role in induction of T cell tolerance by donor-specific transfusion (DST) and hepatocyte transplantation (Wang et al., 2007a, Wang et al., 2014; Zhang et al., 2016). Thus, transgenic expression of human CD47 in pigs may also attenuate xenoimmune responses by ameliorating DC activation and antigen presentation. More recently, transgenic overexpression of CD47 was also applied for reducing allogenicity and generating hypoimmunogenic pluripotent stem cells (Han et al., 2019; Deuse et al., 2019).

It has become increasingly evident that the CD47-SIRPα pathway plays a critical role in containing anti-tumor immune responses. CD47 upregulation was detected in various cancer cells, serving a powerful mechanism of evading macrophage killing (Jaiswal et al., 2009; Chan et al., 2009). Accordingly, treatment with CD47 blockade could inhibit tumor growth via macrophage-mediated mechanism (Jaiswal et al., 2009; Willingham et al., 2012; Weiskopf et al., 2016; Chao et al., 2010; Liu et al., 2015a). More recently, the antitumor activity of CD47 blockade was found to be associated with CD11c^+^ DC activation and largely T cell-dependent (Liu et al., 2015b, Chen et al., 2020; Li et al., 2020). Taken together, these studies revealed clearly that the CD47-SIRPα pathway provides a powerful negative regulation for both innate and adaptive immune responses and is increasingly considered as an effective intervention target for protecting against transplant rejection and unleashing immune responses to cancer.

Although negative regulation of immune responses is predominantly mediated by inhibitory CD47-SIRPα signaling in macrophages and DCs, it remains largely unknown how transgenic CD47 on pig or human pluripotent stem cells and upregulated CD47 on tumor cells interact with its receptor and ligands. In the present study, we identified an alternative source of cell surface CD47. We found that extracellular vesicles (EVs), including exosomes (Exos) from cells transgenically overexpressing CD47 or tumor cells overexpressing endogenous CD47, could induce CD47 cross-dressing on pig or human cells. CD47 cross-dressed on cell surfaces can interact with SIRPα to inhibit phagocytosis. However, unlike the autogenous CD47 that, upon ligation, induces cell apoptosis and senescence (Mateo et al., 1999; Gao et al., 2016; Meijles et al., 2017), ligation of CD47 cross-dressed on cell surfaces is not harmful to cells. This study provides deeper insight into the effect of CD47 overexpression, which needs to be considered when designing strategies for gene-editing in pigs for xenotransplantation or in human pluripotent stem cells for cell replacement therapy, and for developing CD47 blockade-based cancer immunotherapy.

## Results

### Transgenic hCD47 cross-dressing in pig cells

**Figure 1.**
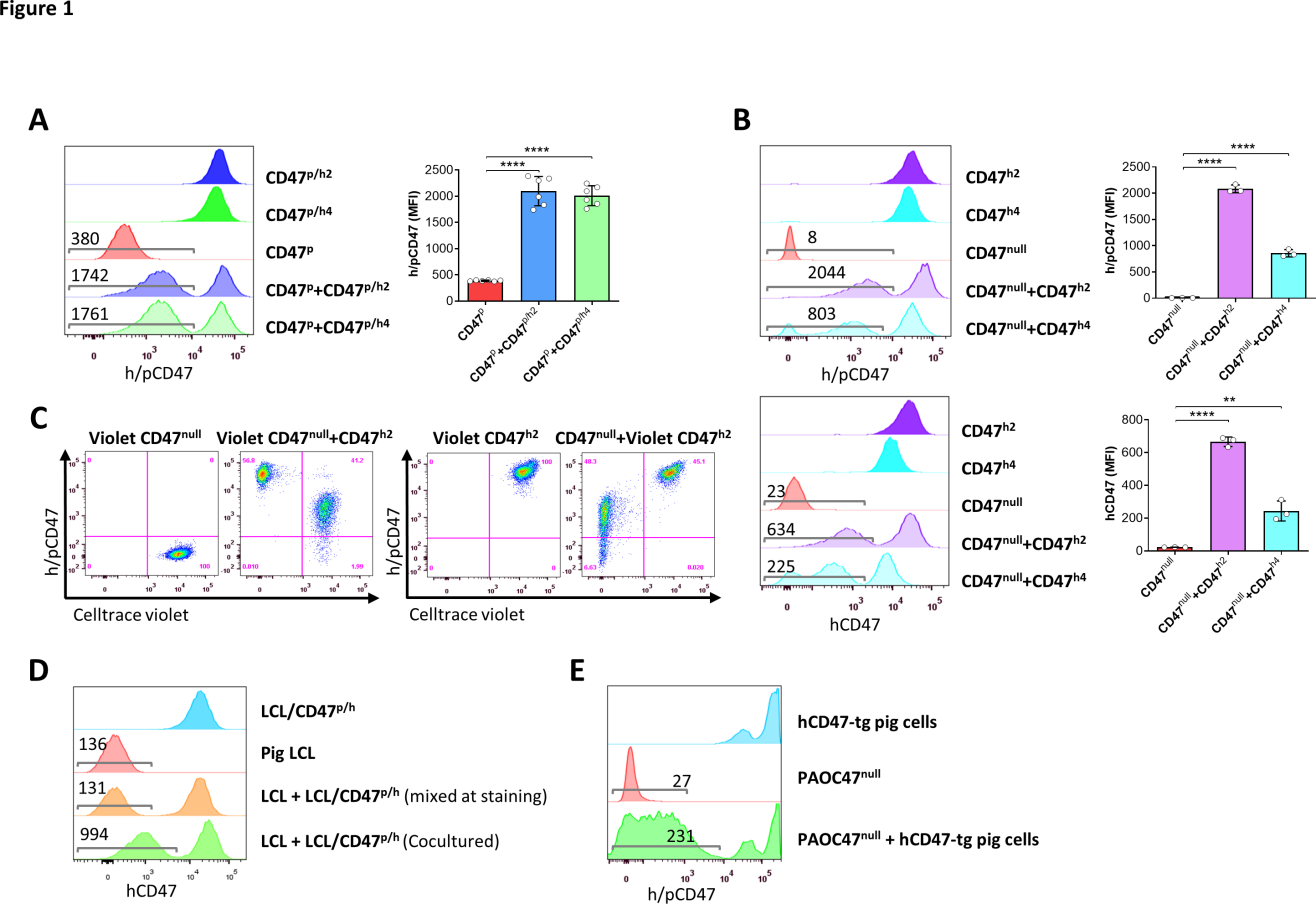
Transgenic hCD47 cross-dressing on pig cells. (**A**) Porcine aortic cell (PAOC)/CD47^p^ cells and PAOC/CD47^p/h2^ or PAOC/CD47^p/h4^ cells were cultured alone or cocultured for 24 hr, and hCD47 cross-dressing on gated PAOC/CD47^p^ cells was assessed by flow cytometry using anti-h/pCD47-PE mAb (clone CC2C6, reacting with both human and pig CD47). Shown are representative histogram profiles (left; the numbers in the figure indicate the median fluorescent intensity [MFI] of gated PAOC/CD47^p^ cells), and average MFI (right; mean ± SDs; n=6 replicates per group) of gated PAOC/CD47^p^ cells in the indicated cell cultures. ****, p<0.0001 (two-tailed unpaired t-test). Results shown are representative of three independent experiments. (**B**) PAOC/CD47^null^, and PAOC/CD47^h2^ or PAOC/CD47^h4^ were cultured alone or cocultured for 24 hr, and analyzed for hCD47 cross-dressing on gated PAOC/CD47^null^ cells by flow cytometry using anti-h/pCD47-PE mAb (*top*) or anti-hCD47-BV786 mAb (*bottom*). Shown are representative histogram profiles (left panel; the numbers in the figure indicate the MFI of gated PAOC/CD47^null^ cells), and average MFI (right panel; mean ± SDs; n=3 replicates per group) of gated PAOC/CD47^null^ cells in the indicated cell cultures. **, p<0.01; ****, p<0.0001 (two-tailed unpaired t-test). Results shown are representative of three independent experiments. (**C**) Celltrace violet-labeled PAOC/CD47^null^ (left panel) or PAOC/CD47^h2^ (right panel) was cultured alone (Violet CD47^null^ or Violet CD47^h2^) or cocultured with unlabeled PAOC/CD47^h2^ (Violet CD47^null^ + CD47^h2^) or PAOC/CD47^null^ (CD47^null^ or Violet CD47^h2^), respectively, then the cells were stained by anti-h/pCD47-PE mAb. Shown are representative flow cytometry profiles (n=3 replicates). Results shown are representative of two independent experiments. (**D**) Pig lymphoma cell line (LCL) and hCD47-tg LCL (LCL/CD47^p/h^) cells were cultured alone or cocultured for 24 hr, and analyzed for hCD47 cross-dressing on gated LCL cells (the numbers in the figure indicate the MFI of gated LCL cells). The staining control of ‘mixed at staining’ indicates the two types of cells were cultured separately and mixed immediately prior anti-CD47 staining. Two independent experiments were performed, and each experiment had two replicates per group. Representative flow cytometry profiles are shown. (**E**) PAOC47^null^ cells were cocultured with bone marrow cells from hCD47-tg miniature swine for 2 days and analyzed for hCD47 cross-dressing on gated PAOC47^null^ cells by flow cytometry using anti-h/pCD47-PE mAb (the numbers in the figure indicate the MFI of gated PAOC/CD47^null^ cells). Two independent experiments were performed, and each experiment had two replicates per group. Representative flow cytometry profiles are shown.

CD47 cross-dressing was first identified by detecting hCD47 on pig cells that was cocultured with pig cells expressing transgenic hCD47. Two different hCD47 isoforms were used to ensure that findings are not isoform-specific. In these experiments, cell cocultures were performed using cell line cells derived from porcine aortic cells (PAOC; Figure 1—figure supplement 1). First, we cocultured parental PAOCs (expressing pig CD47; referred to as PAOC/CD47^p^) with PAOCs that were genetically modified to express hCD47 isoform 2 (referred to as PAOC/CD47^p/h2^) or isoform 4 (referred to as PAOC/CD47^p/h4^). Two different CD47 isoforms were used to confirm the findings are not isoform-specific. Flow cytometry analysis using anti-CD47 antibodies recognizing both human and pig CD47 revealed that PAOC/CD47^p/h2^ or PAOC/CD47^p/h4^ cells expressed a markedly increased level of CD47 compared to PAOC/CD47^p^ cells, and that PAOC/CD47^p^ cells showed significantly increased CD47 staining after coculture with PAOC/CD47^p/h2^ or PAOC/CD47^p/h4^ (Figure 1A). These results suggest that PAOC/CD47^p^ cells were cross-dressed by CD47, likely transgenic hCD47, from PAOC/CD47^p/h^ cells during cultures. To confirm this possibility, we made CD47-defficient PAOC cells (via targeted deletion using CRISPR-Cas9- technology; referred to as PAOC/CD47^null^) and PAOC47^null^ cells that express transgenic hCD47 isoform 2 (PAOC/CD47^h2^) or isoform 4 (PAOC/CD47^h4^). When the PAOC cell line cells were cocultured for 24 hr, we found that PAOC47^null^ cells became positively stained by both anti-h/pCD47 (Figure 1B**, top**) and anti-hCD47 (Figure 1B**, bottom**) antibodies. To rule out the possibility that, during the coculture, the CD47^null^ cells did not become CD47^+^, but PAOC/CD47^h2^ cells reduced hCD47 expression, we labeled PAOC/CD47^null^ (Figure 1C**, Left**) or PAOC/CD47^h2^ (Figure 1C**, Right**) cells with florescence Celltrace violet and then cocultured the labeled cells with unlabeled PAOC/CD47^h2^ or PAOC/CD47^null^ cells, respectively. This experiment, in which fluorescence-labeling allowed for better distinguishing between the two cell populations in the cocultures, further confirmed that PAOC/CD47^null^ cells can be cross-dressed by CD47 after coculture with PAOC/CD47^h2^ cells (Figure 1C).

**Figure 1—figure supplement 1.**
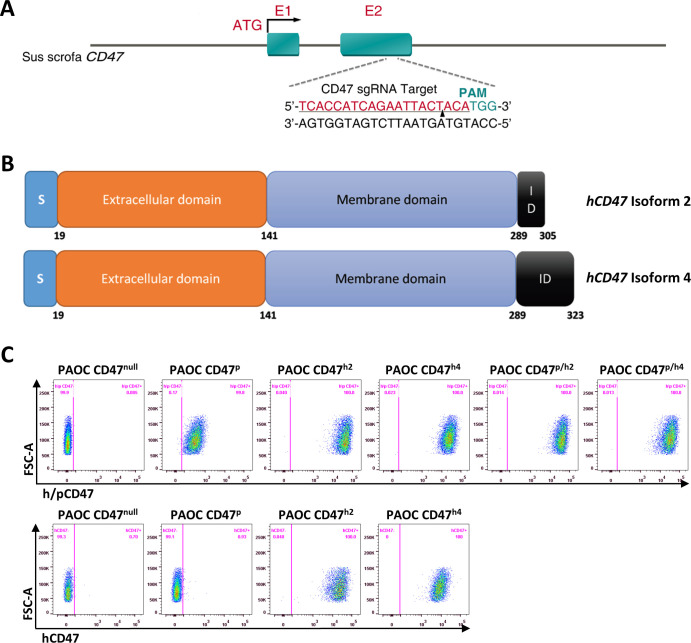
Generation of CD47-deficient and hCD47-trangeneic porcine aortic cell (PAOC) cell lines. (**A**) Schematic of pig CD47 loci and guide RNA sequence targeting the exon 2 of pig CD47 gene used for generating POAC/CD47^null^ cells. (**B**) Schematics of human CD47 isoform 2 (with 16aa intracellular domain (ID); top) and isoform 4 (with 34aa ID; bottom) plasmids, which were used for making POAC/CD47^h2^ and POAC/CD47^h4^ cells, respectively. (**C**) flow cytometry profiles showing staining of the indicated PAOC cell lines with anti-h/p CD47 (top) and anti-hCD47 (bottom) antibodies.

**Figure 1—figure supplement 2.**
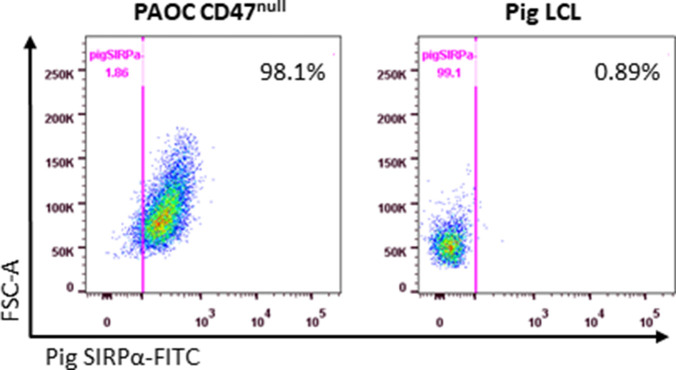
SIRPα expression on pig cells. Shown are representative flow cytometry profiles of porcine aortic cell (PAOC) CD47^null^ (left) and pig lymphoma cell line (LCL; right) cells stained with anti-pig SIRPα.

PAOC cells express SIRPα (Figure 1—figure supplement 2**, left**), and pig SIRPα is reported to interact with human CD47 (Boettcher et al., 2019). Furthermore, previous studies have shown that CD47-lentiviruses are more effective in transducing SIRPα^+^ non-phagocytic tumor cells, suggesting that the interaction of CD47 with SIRPα may facilitate lentiviral engulfment by target cells (Sosale et al., 2016). Thus, to determine whether hCD47 cross-dressing is mediated by binding of hCD47 to pig SIRPα, we performed cocultures with pig lymphoma cell line (LCL) cells that do not express SIRPα (Figure 1—figure supplement 2**, right**). LCL cells cocultured with LCL cells that express hCD47 (LCL/CD47^p/h^) (Ide et al., 2007; Wang et al., 2011), but not those cultured alone or mixed with LCL/CD47^p/h^ immediately prior to flow cytometry analysis, were positively stained by anti-hCD47 antibodies (Figure 1D). Although we cannot rule out of the role of SIRPα in hCD47 cross-dressing, our data indicate that hCD47 cross-dressing can occur in an SIRPα-independent manner.

We then wished to determine if cells other than the PAOC cell lines could be a source of CD47 for cross-dressing. Toward this end, PAOC47^null^ cells were cocultured with bone marrow cells (BMCs) from hCD47-transgenic miniature swine. Like pig cells cocultured with hCD47-transgenic cell lines (Figure 1A–D), PAOC47^null^ cells also became positively stained by anti-hCD47 antibodies after being cocultured with hCD47-tg swine cells (Figure 1E).

### Cross-dressing with native CD47 from human T cell leukemia cells

**Figure 2.**
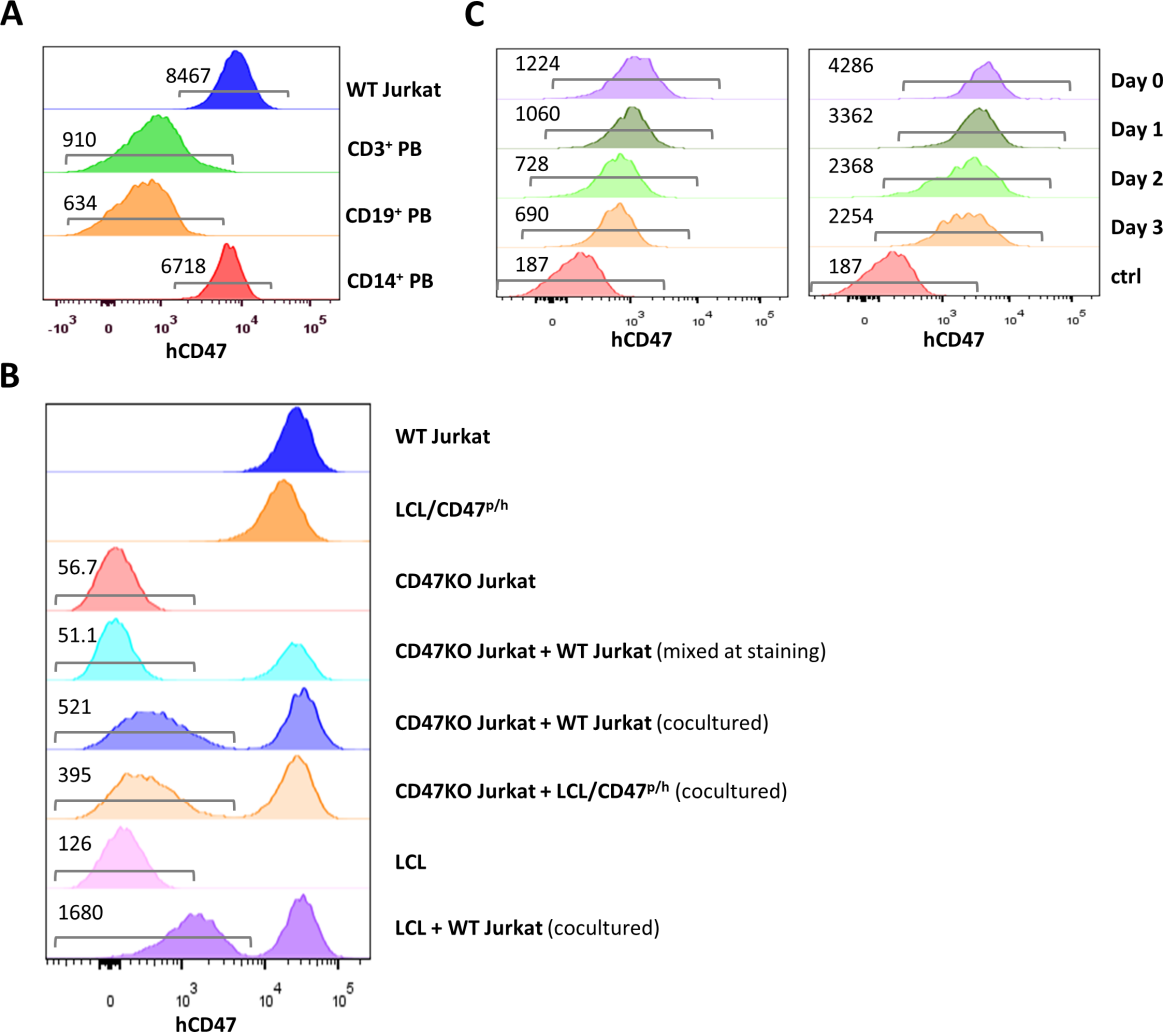
CD47 expression and CD47 cross-dressing on human T cell leukemia Jurkat cells. (**A**) CD47 expression on Jurkat cells and normal human CD3^+^, CD19^+^, and CD14^+^ peripheral blood cells (the numbers indicate median fluorescent intensity [MFI] of human CD47 staining). (**B**) CD47 expression on wild-type (WT) Jurkat cells, pig lymphoma cell line (LCL)/CD47^p/h^ cells, CD47KO Jurkat cells, CD47KO cells mixed with WT Jurkat cells (mixed at the time of staining), CD47KO Jurkat cells cocultured (24 hr) with WT Jurkat or pig LCL/CD47^p/h^ cells, pig LCL cells, and LCL cells cocultured (24 hr) with WT Jurkat cells. (**C**) PKH67-labeled pig LCL cells were cocultured with PHK26-labeled LCL/CD47^p/h^ (left) or WT Jurkat (right) cells for 1 day, PHK67-labeled pig LCL cells are sorted and treated with mitomycin C (2 µg/ml) for 30 min (to stop cell division), then analyzed for hCD47 staining on pig LCL cells immediately (Day 0) and at the indicated times after cultured in media. The numbers in the figure indicate MFI of CD47 staining on gated cells.

We next determined whether cells can be cross-dressed by native CD47 using human T-cell leukemia Jurkat cells that express a higher level of CD47 than normal hematopoietic cells (Figure 2A). In order to clearly identify cross-dressed CD47 on cell surface, CD47-defficient Jurkat cells were generated using the CRISPR-Cas9 technique (Figure 2—figure supplement 1) and cocultured for 24 hr with the parental WT Jurkat cells or with hCD47-tg pig LCL cells (Figure 2B). Flow cytometry analysis revealed that CD47KO Jurkat cells were clearly stained positive by anti-hCD47 antibodies after coculture with parental WT Jurkat cells or hCD47-tg pig LCL cells compared to those cultured alone or mixed immediately before staining (Figure 2B). Furthermore, pig LCL cells also became positive for human CD47 staining after coculture for 24 hr with WT Jurkat cells (Figure 2B). More than half of the cross-dressed hCD47 (determined by MFI) remained on pig LCL cells 3 days after being separated from hCD47-expressing cells (Figure 2C), indicating that hCD47 cross-dressing was relatively stable. We also noted that not only hCD47, but other membrane proteins of WT Jurkat cells were also cross-dressed on pig LCL cells (Figure 2—figure supplement 2). While to a lower level than untreated cells, hCD47 cross-dressing was clearly detected on LCL cells that were pretreated with cytochalasin D, a cell-permeable potent inhibitor that disrupts actin microfilaments (Cooper, 1987), after cocultured with cytochalasin D-pretreated WT Jurkat cells (Figure 2—figure supplement 3A), indicating that a functional cytoskeleton is not absolutely required for, but may facilitate hCD47 cross-dressing. These results indicate that human CD47 cross-dressing could be induced by not only hCD47-transgenic cells but also tumor cells that express only the native CD47.

**Figure 2—figure supplement 1.**
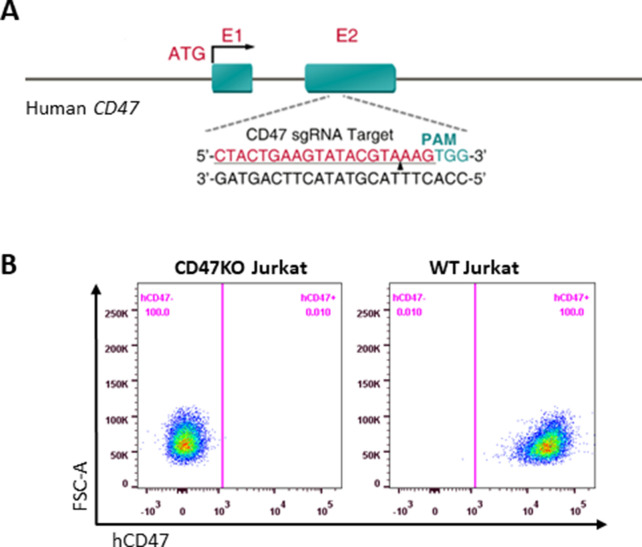
Generation of CD47-deficient Jurkat cells. (**A**) Schematic of human CD47 loci and guide RNA sequence targeting the exon 2 of human CD47 gene used for generating CD47KO Jurkat cells. (**B**) Flow cytometry profiles showing anti-CD47 staining of wild-type (WT) and CD47KO Jurkat cells.

**Figure 2—figure supplement 2.**
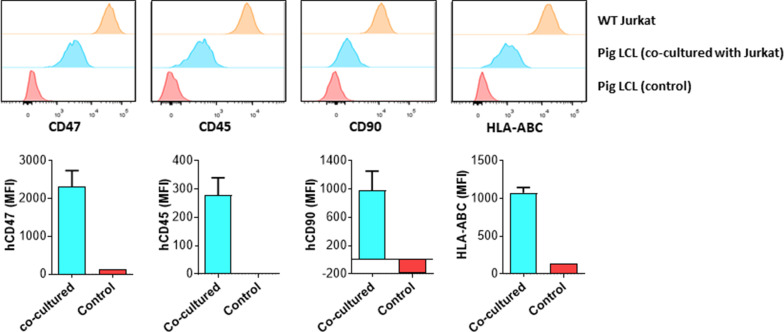
Multiple membrane proteins are cross-dressed during cell coculture. PKH67-labeled pig lymphoma cell line (LCL) cells were cocultured with PKH26 labeled-wild-type (WT) Jurkat cells for 20 hr and analyzed for human CD47, CD45, CD90, and HLA-ABC staining on pig LCL cells. Shown are representative histogram, and MFI (mean ± SDs; n = 3 replicates in the co-cultured group) of pig LCL cells co-cultured with WT Jurkat cells. Results shown are representative of three independent experiments.

**Figure 2—figure supplement 3.**
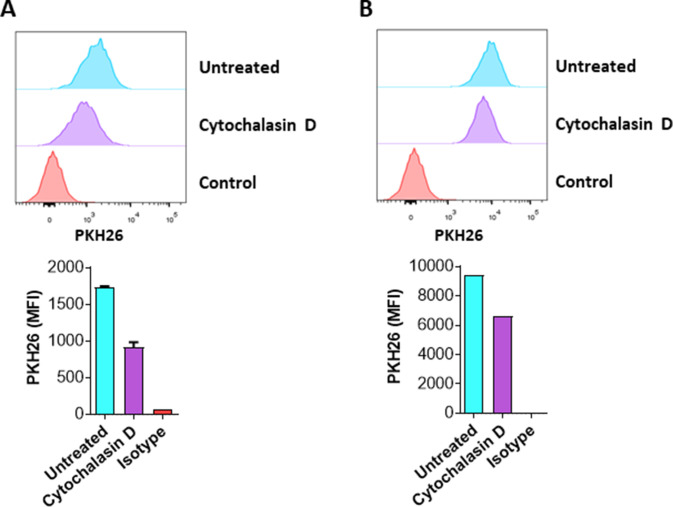
A functional cytoskeleton is not absolutely required for but may facilitate hCD47 cross-dressing. Untreated or cytochalasin D-pretreated pig lymphoma cell line (LCL) cells were cocultured with PKH26-labeled wild-type Jurkat cells (**A**) or extracellular vesicles (prepared from LCL/CD47^p/h^) (**B**) for 20 hr, and analyzed for PKH26 fluorescence on pig LCL cells by flow cytometry. MFI: median fluorescent intensity. Results shown are representative of two independent experiments.

### CD47 cross-dressing by extracellular vesicles and exosomes

**Figure 3.**
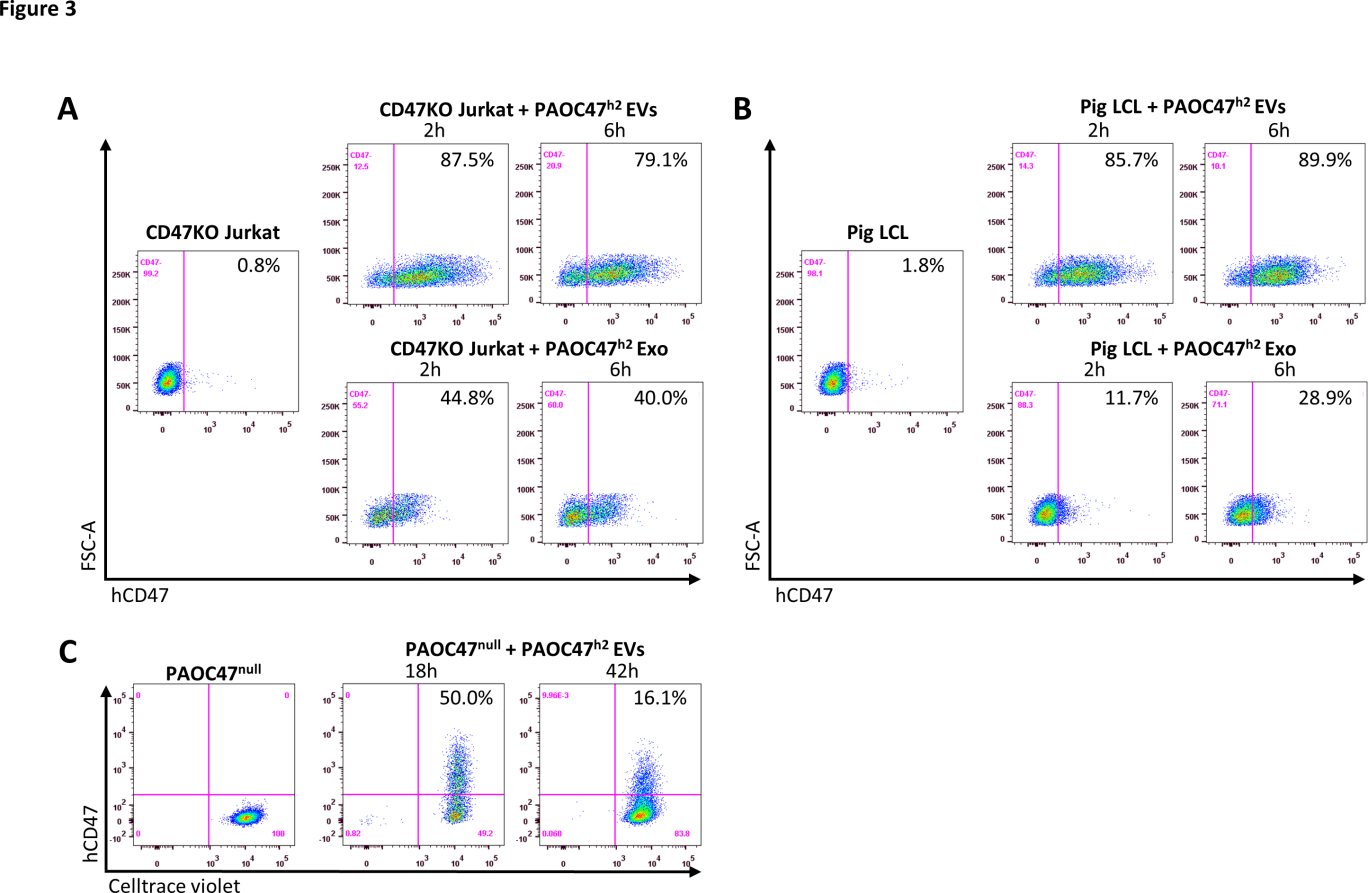
CD47 cross-dressing by extracellular vesicles (EVs) and exosomes (Exos). (**A–B**) CD47KO Jurkat cells (**A**) or pig lymphoma cell line (LCL) cells (**B**) were cultured in the absence (*left*) or presence (*right*) of EVs (*top*) or Exos (*bottom*) prepared from PAOC/CD47^h2^ cell culture supernatants for 2 hr or 6 hr, and analyzed for hCD47 cross-dressing by flow cytometry using anti-hCD47-BV786 mAb. Representative flow cytometry profiles of three independent experiments were shown. (**C**) Celltrace violet-labeled PAOC/CD47^null^ cells were cultured in the absence (*left*) or presence (*right*) of EVs prepared from PAOC/CD47^h2^ cells for 18 hr or 42 hr, and analyzed for hCD47 cross-dressing by flow cytometry using anti-hCD47-BV786 mAb.

We next investigated whether CD47 cross-dressing can be induced by EVs or requires direct cell-cell interaction. We analyzed hCD47 cross-dressing on CD47KO Jurkat cells, pig LCL cells, and PAOC/CD47^null^ cells in the absence or presence of EVs prepared from PAOC/CD47^h2^ cells. Flow cytometry analysis showed that CD47 cross-dressing occurred in both CD47KO human T-cell leukemia Jurkat cells (Figure 3A) and pig B-lymphoma LCL cells (Figure 3B) after incubation for 2 or 6 hr with PAOC47^h2^ cell-derived EVs. To a less extent, both CD47KO Jurkat and LCL cells were also positively stained by anti-hCD47 after incubation with Exos released by PAOC47^h2^ cells (Figure 3A and B). Similarly, hCD47 cross-dressing was detected in PAOC^null^ cells after incubation with EVs from PAOC47^h2^ cells (Figure 3C). In this experiment, PAOC^null^ cells were labeled with florescence Celltrace violet prior to incubation with EVs to ensure there was no contamination by PAOC^h2^ cells in the prepared EVs. After incubation with PAOC47^h2^ EVs, a significant proportion of violet-labeled PAOC^null^ cells became positive for hCD47 (Figure 3C). Of note, the frequency of hCD47^+^ PAOC^null^ cells at 42 hr was lower than that at 18 hr, which is most likely due to PAOC^null^ cell proliferation and EV exhaustion/degradation. Pig LCL cells were also stained positive for hCD47 after incubated with EVs from hCD47-tg pig LCL cells or WT Jurkat cells, in which hCD47 cross-dressing was detected 1 hr after incubation with EVs and the levels remained stable or increased at 24 hr (Figure 3—figure supplement 1). These results indicate that CD47 cross-dressing could be induced independently of cell-cell contact by EVs, including Exos. Again, a functional cytoskeleton is not absolutely required for hCD47 cross-dressing, as hCD47 was clearly detected on cytochalasin D-pretreated LCL cells after incubation with EVs from hCD47-tg LCL cells, though the level was moderately reduced (Figure 2—figure supplement 3B). Furthermore, cross-dressing was not diminished by treatment with trypsin/EDTA, indicating that membrane fusion is involved in EV cross-dressing (Figure 3—figure supplement 2).

**Figure 3—figure supplement 1.**
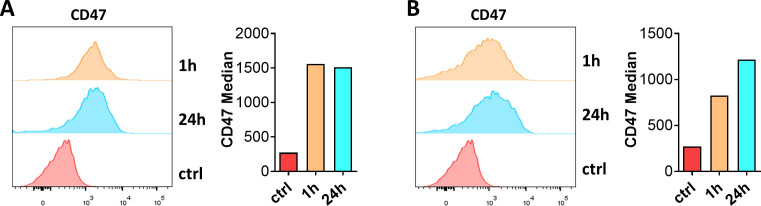
Rapid CD47 cross-dressing on pig lymphoma cell line (LCL) cells incubated with CD47-expressing extracellular vesicles (EVs). Pig LCL cells were cultured with EVs from hCD47-tg pig LCL (**A**) or wild-type Jurkat cells (**B**), and analyzed for CD47 cross-dressing by flow cytometry at 1 and 24 hr after culture. Pig LCL cells that were not cultured with EVs were used as the staining control. Results shown are representative of three independent experiments.

**Figure 3—figure supplement 2.**
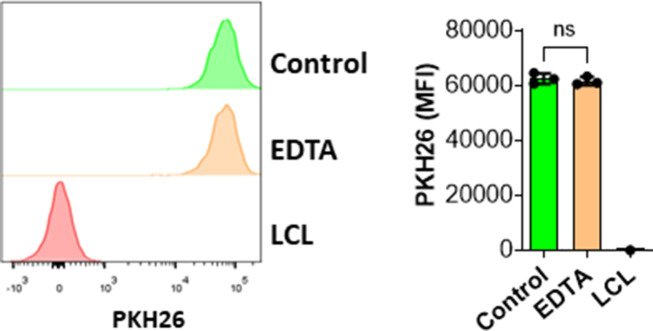
Cross-dressing involves membrane fusion. Wild-type Jurkat cells were labeled with PKH26 and cocultured with PKH67-labeled pig lymphoma cell line (LCL) cells for 24 hr, washed with PBS with or without trypsin/EDTA, then analyzed PKH26 fluorescence on LCL cells by flow cytometry. Shown are staining profiles (left) and median fluorescent intensity (MFI; mean ± SDs) of cocultured LCL cells that were not (Control, n=3) or treated with trypsin/EDTA (EDTA,n=3), ns, not significant (two-tailed unpaired t-test); LCL cells not cocultured with Jurkat cells were used as staining control (LCL). Results shown are representative of two independent experiments.

### Protection against phagocytosis by cross-dressed CD47

**Figure 4.**
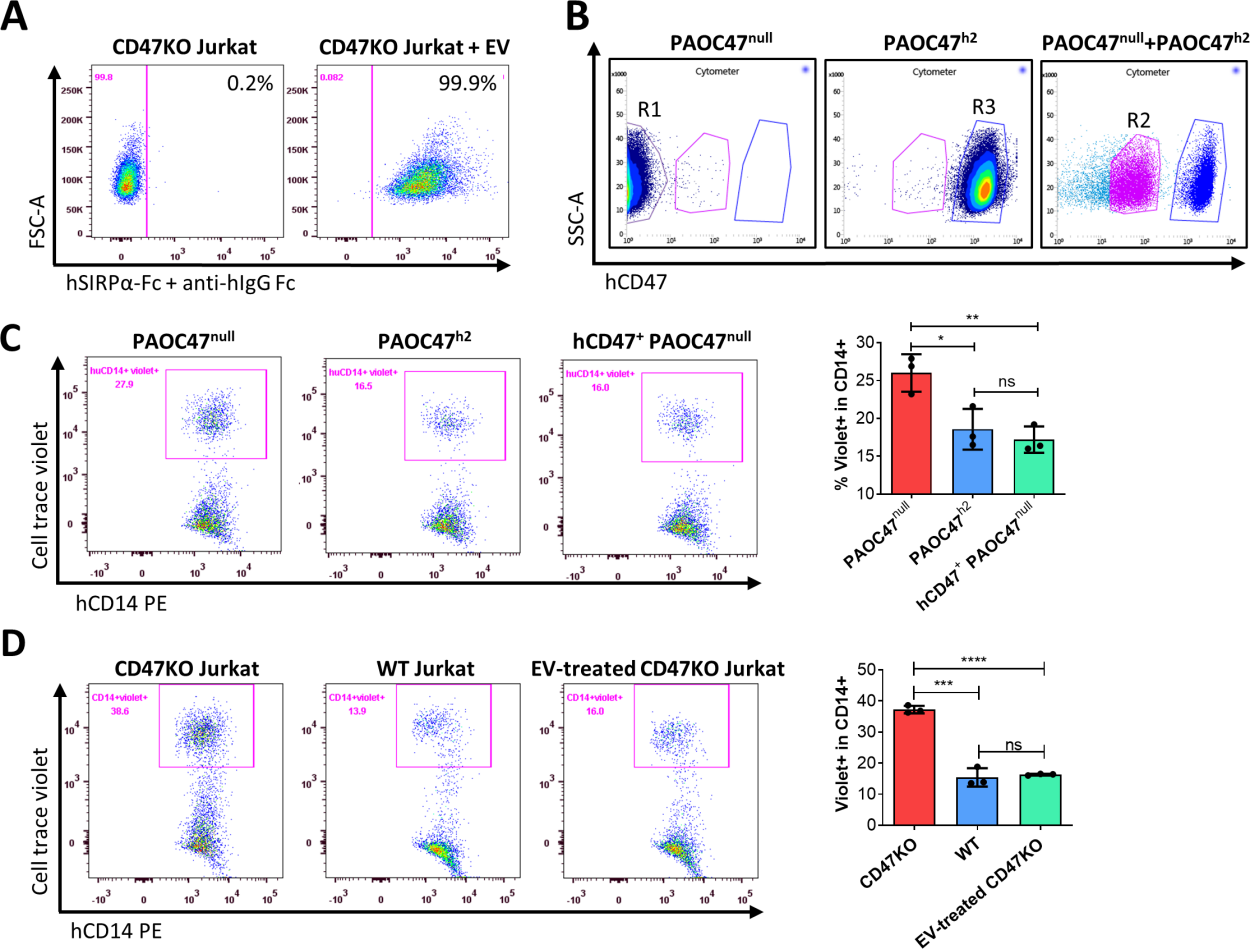
Protection against phagocytosis by cross-dressed CD47. (**A**) CD47KO Jurkat cells were cocultured without (*left*) or with (*right*) extracellular vesicles (EVs) from PAOC/CD47^h2^ cells at 37°C for 5 hr, then washed and incubated with recombinant human SIRPα-Fc chimera at 37°C for 1 hr. The binding of SIRPα-Fc proteins to CD47KO Jurkat cells was visualized by staining with APC-conjugated mouse anti-human IgG Fc mAb. Representative flow cytometry profiles of two independent experiments are shown. (**B**) PAOC47^null^ and PAOC47^h2^ cells were cultured alone (*left* and *middle*) or together (*right*) for 48 hr and stained using anti-hCD47-BV786 mAb, then PAOC47^null^ (**R1**), PAOC47^h2^ (**R3**), and hCD47^+^ (i.e. hCD47 cross-dressed) PAOC47^null^ (R2) cells sorted from cocultures were used immediately for phagocytic assay. (**C**) PAOC47^null^ (**R1**), PAOC47^h2^ (**R3**), or sorted hCD47 cross-dressed PAOC47^null^ (R2) cells were labeled with Celltrace violet and incubated with human macrophages for 2 hr, then phagocytosis was determined by flow cytometry using anti-human CD14 mAb. Shown are representative flow cytometry profiles (left) and levels (right; mean ± SDs; n=3) of phagocytosis (i.e. percentages of human macrophages that have engulfed violet + target cells (CD14^+^violet^+^) in human CD14^+^ macrophages). Representative results of three independent experiments are shown. (**D**) CD47KO Jurkat, wild-type (WT) Jurkat, and CD47KO Jurkat cells pre-incubated with EVs from PAOC/CD47^h2^ cells were labeled by Celltrace violet and cocultured with human macrophages for 2 hr, then phagocytosis was analyzed by flow cytometry. Shown are representative flow cytometry profiles (left) and levels (right; mean ± SDs; n=3) of phagocytosis (i.e. percentages of hCD14^+^violet^+^ in total hCD14^+^ macrophages). Representative results of two independent experiments are shown. *, p<0.05; **, p<0.01; ***, p<0.001; ****, p<0.0001; ns, not significant (two-tailed unpaired t-test).

We next determined whether cross-dressed CD47 can act as a marker of self to protect the cells against phagocytosis. We first investigated the binding potential of cross-dressed hCD47 with human SIRPα. CD47KO Jurkat cells were cocultured without or with EVs from PAOC/CD47^h2^ cells for 5 hr, washed, and incubated with recombinant human SIRPα-Fc chimera for 1 hr. Binding of human SIRPα fusion protein to CD47KO Jurkat cells was then measured using fluorochrome-labeled anti-human IgG Fc antibody. Flow cytometry analysis showed that human SIRPα fusion protein was able to bind CD47KO Jurkat cells cultured with EVs but not those cultured without EVs (Figure 4A). The data indicate that cross-dressed hCD47 on CD47KO Jurkat cells can bind human SIRPα.

We then performed phagocytic assay to determine the potential of cross-dressed hCD47 to protect pig cells or CD47KO human leukemia cells against phagocytosis by human monocyte-derived macrophages. PAOC47^null^ and PAOC/CD47^h2^ cells were cultured for 48 hr, then hCD47 cross-dressed PAOC47^null^ cells were sorted out (Figure 4B) and their susceptibility to phagocytosis by human macrophages was determined in comparison to PAOC47^null^ and PAOC/CD47^h2^ cells that were cultured separately (Figure 4C). As expected, PAOC47^null^ cells were significantly more sensitive than PAOC/CD47^h2^ cells to phagocytosis (Figure 4C). However, hCD47 cross-dressing effectively reduced the susceptibility of PAOC47^null^ cells to phagocytosis by human macrophages, to a level comparable to that of PAOC/CD47^h2^ cells (Figure 4C). Human CD47 cross-dressing protects not only xenogeneic pig cells, but also human leukemia cells, against phagocytosis by human macrophages. In phagocytic assays where CD47KO Jurkat cells showed significantly greater phagocytosis than WT Jurkat cells, pre-incubation of CD47KO Jurkat cells with EVs released by PAOC/CD47^h2^ cells was found highly effective in reducing their phagocytosis by human macrophages (Figure 4D). These results indicate that human CD47 cross-dressing can act as a functional ligand for human SIRPα and deliver ‘don’t eat me’ signals to human macrophages.

### Ligation of autogenous but not cross-dressed CD47 induces death in Jurkat cells

**Figure 5.**
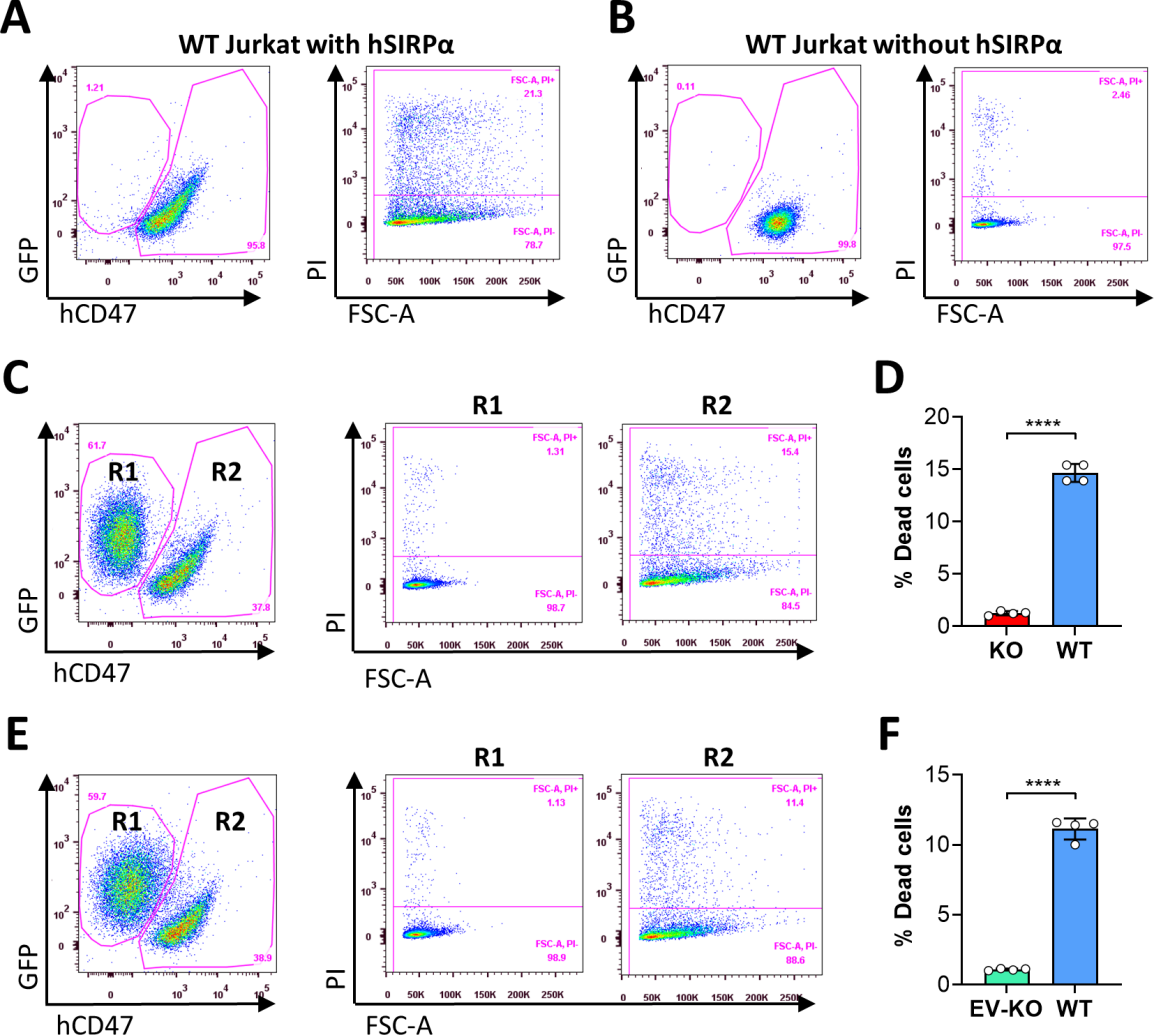
Ligation of autogenous but not cross-dressed CD47 induces cell death. (**A–B**) 5×10^4^ wild-type (WT) Jurkat cells (GFP^−^) were incubated in the presence (**A**) or absence (**B**) of 2.5 µg/ml hSIRPα-Fc proteins at 37°C for 1 hr and stained with anti-hCD47 mAb and Propidium Iodide (PI). Representative flow cytometry profiles show dead cells (PI^+^) in hCD47^+^ WT Jurkat population. (**C–D**) CD47KO Jurkat (GFP^+^) and WT Jurkat cells were mixed (at 1:1 ratio; 5×10^4^ each) and cocultured in the presence of 2.5 µg/ml human SIRPα-Fc for 1 hr, then stained with anti-hCD47 mAb and PI. Shown are representative flow cytometry profiles (**C**) and percentages (D; mean ± SDs) of PI^+^ dead cells in gated CD47KO (R1, CD47^-^GFP^+^) and WT (R2, hCD47^+^GFP^−^) Jurkat cells. (**E–F**) CD47KO Jurkat (GFP^+^) cells were incubated for 2 hr with PAOC/CD47^h2^ extracellular vesicles (EVs), then mixed with WT Jurkat cells (at 1:1 ratio; 5×10^4^ each) and cocultured in the presence of 2.5 µg/ml human SIRPα-Fc for 1 hr. The cells were stained with anti-hCD47 mAb and PI. Shown are representative flow cytometry profiles (**E**) and percentages (F; mean ± SDs) of PI^+^ dead cells in gated EV-treated CD47KO (R1, CD47^low^GFP^+^) and WT (R2, hCD47^+^GFP^-^) Jurkat cells. ****, p<0.0001 (two-tailed unpaired t-test). Of note, the cells cultured with hSIRPα-Fc proteins (**A, C and E**) showed reduced hCD47 staining (as detailed in Figure 5—figure supplement 2). Results shown are representative of three independent experiments.

Ligation of cell surface CD47 by its ligand thrombospondin-1 (TSP-1) (Saumet et al., 2005), CD47-binding peptides of TSP-1 (Martinez-Torres et al., 2015) or CD47 antibodies (Mateo et al., 1999) has been shown to induce death in varying types of cells. In line with these reports, we observed that human SIRPα-Fc fusion proteins could induce cell death in a dose-dependent manner in WT, but not CD47KO, human T-cell leukemia Jurkat cells (Figure 5—figure supplement 1; Figure 5A and B). The cell death observed in WT Jurkat cells was induced by CD47 ligation with hSIRPα-Fc proteins, as cell death was minimally detectable in WT Jurkat cells that were cultured simultaneously without hSIRPα-Fc proteins. To determine whether cross-dressed CD47 on Jurkat cells may also induce cell death, we compared the susceptibility to cell death induced by SIRPα-Fc proteins among WT, CD47KO, and hCD47 cross-dressed CD47KO Jurkat cells. CD47 cross-dressing was performed on GFP^+^ CD47KO Jurkat cells by incubation for 2 hr with PAOC/CD47^h2^ EVs. To induce cell death, GFP^+^ CD47KO or hCD47 cross-dressed GFP^+^ CD47KO Jurkat cells were cocultured, respectively, with an equal number of control WT Jurkat cells (5×10^4^ each) in the presence of human SIRPα-Fc for 1 hr. The cocultured cells were then stained with anti-hCD47 (BV786) mAb, and cell death in WT (hCD47^+^GFP^-^), CD47KO (CD47^-^GFP^+^), and hCD47 cross-dressed CD47KO (CD47^low^GFP^+^) Jurkat cells were measured. Of note, binding of hSIRPα-Fc proteins to cell surface CD47 (either native or cross-dressed) could partially block subsequent staining with anti-hCD47 antibodies and thus, the cells cultured with hSIRPα-Fc showed relatively lower hCD47 staining than those cultured without (Figure 5—figure supplement 2, Figure 5). We found that SIRPα-Fc proteins induced significant cell death in WT Jurkat cells regardless of whether they were cocultured with CD47KO (Figure 5C and D) or with hCD47 cross-dressed CD47KO (Figure 5E and F). CD47 cross-dressing did not increase the sensitivity to cell death induced by SIRPα-Fc proteins, and cell death was minimally detectable in both CD47KO (Figure 5C and D) and hCD47 cross-dressed CD47KO (Figure 5E and F) Jurkat cells. However, CD47KO Jurkat cells regained the sensitivity to apoptosis induced by CD47 agonists after being transfected to express CD47 (Figure 5—figure supplement 3). These results indicate that, unlike autogenous CD47, cross-dressed CD47 on Jurkat cells does not induce cell death upon ligation with SIRPα-Fc proteins.

**Figure 5—figure supplement 1.**
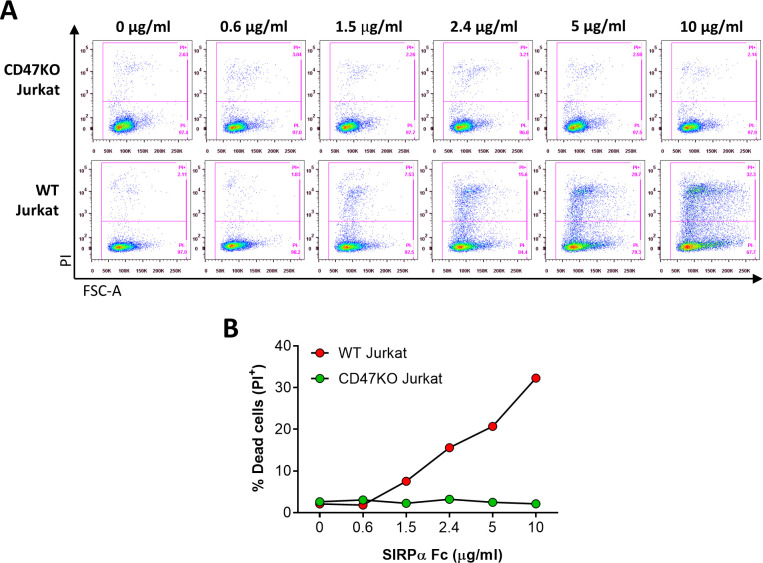
SIRPα-Fc-induced cell death in wild-type (WT) and CD47KO Jurkat cells. 1×10^5^ CD47KO or WT Jurkat cells were incubated in with hSIRPα-Fc proteins at the indicated concentrations for 1 hr at 37°C, then stained with PI to identify dead cells. (**A**) Representative flow cytometry profiles. (**B**) Percentages of dead (PI^+^) cells. Representative results of two independent experiments are shown.

**Figure 5—figure supplement 2.**
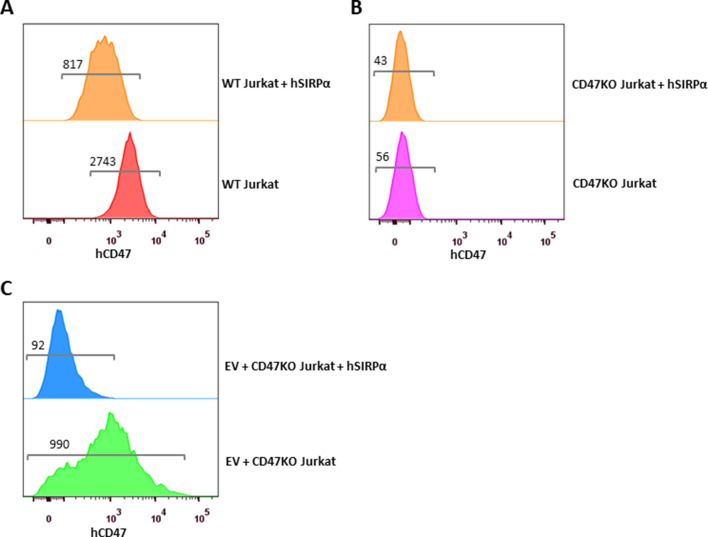
Prior incubation with hSIRPα-Fc proteins partially blocks subsequent staining by anti-hCD47 mAb. (**A**) Anti-hCD47 staining of wild-type (WT) Jurkat cells with or without prior incubation with hSIRPα-Fc. (**B**) Anti-hCD47 staining of CD47KO Jurkat cells with or without prior incubation with hSIRPα-Fc. (**C**) Anti-hCD47 staining of PAOC/CD47^h2^ extracellular vesicle (EV)-treated (for 2 hr) CD47KO Jurkat cells with or without prior incubation with hSIRPα-Fc. The numbers in the figures indicate median fluorescent intensity levels. Results shown are representative of three independent experiments.

**Figure 5—figure supplement 3.**
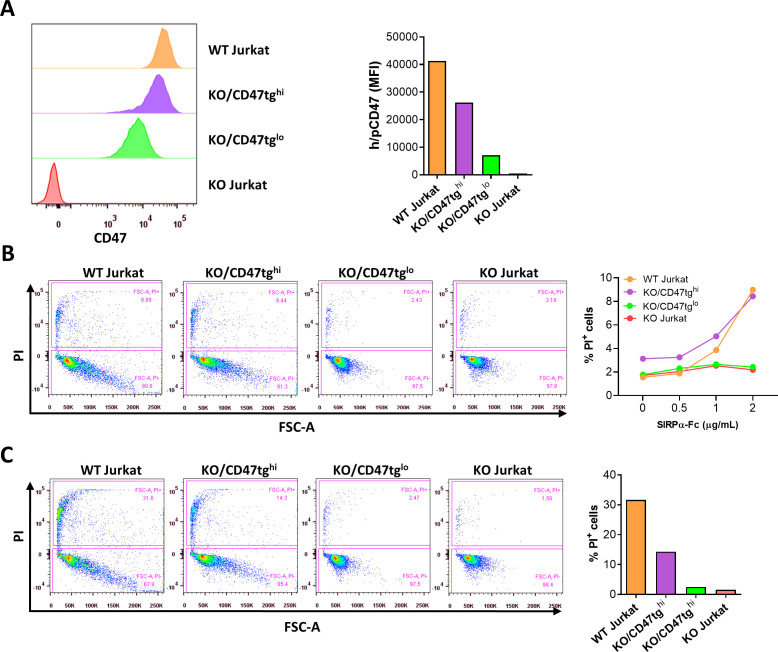
CD47-transfected CD47KO Jurkat cells are sensitive to apoptosis induced by CD47 agonists. (**A**) CD47 expression on wild-type (WT), transfected CD47KO (KO/CD47tg), and control CD47KO Jurkat cells. (**B**) Apoptosis induced by SIRPa-Fc proteins. (**C**) Apoptosis induced by agonistic anti-CD47 antibody CC2C6 (5 µg/ml). Results shown are representative of two independent experiments.

## Discussion

Given its strong inhibitory effects on macrophage activation and phagocytosis, transgenically overexpressed CD47 is considered an effective means of preventing transplant rejection. A major obstacle impeding the translation of xenotransplantation into clinical therapies is vigorous xenograft rejection (Yang and Sykes, 2007), and the lack of functional interaction in CD47-SIRPα pathway is a key mechanism triggering macrophage xenoimmune responses (Yang, 2010). Studies have shown that the use of gene-edited pigs carrying human CD47 is effective in protecting against xenograft rejection by macrophages in non-human primates (Ide et al., 2007; Tena et al., 2017; Watanabe et al., 2020). Recently, transgenic overexpression of CD47 was also successfully used in combination with other approaches, such as deletion of HLA molecules, to generate hypoimmunogenic pluripotent stem cells (Han et al., 2019; Deuse et al., 2019). Here we found that EVs and Exos released from pig cells transgenically overexpressing hCD47 can mediate hCD47 cross-dressing on surrounding pig cells. Furthermore, hCD47 cross-dressed on pig cells can interact with human SIRPα and inhibit phagocytosis by human macrophages. Such CD47 cross-dressing occurs not only in the pig-to-pig combination, but also in the human-to-human, pig-to-human, and human-to-pig directions. These results provide a new mechanism for the inhibition of phagocytosis by the approach of transgenically expressing CD47.

In both xenogeneic and allogeneic settings, a high level of transgenic CD47 expression was found to be essential for its immune inhibitory effect. CD47 is not only a ligand of SIRPα, but also a signaling receptor that mediates a variety of functions, including apoptosis, cell cycle arrest, and senescence. It was reported that deletion of CD47 improves survival, proliferation, and function of endothelial cells, leading to increased angiogenesis and neovascularization both in vitro and in vivo (Meijles et al., 2017; Gao et al., 2016; Gao et al., 2017). In line with these observations, CD47 deletion from organ grafts was reported to ameliorate renal ischemia/reperfusion injury (Isenberg and Roberts, 2019) and cardiac allograft rejection (Chen et al., 2019). Using a pig-to-baboon kidney xenotransplantation model, a recent study suggested that widespread expression of hCD47 in the pig kidney was associated with increased vascular permeability and systemic edema, presumably due to upregulated TSP-1, and hence, TSP-1-CD47 signaling in the graft (Takeuchi et al., 2021). Our study showed that, although CD47 cross-dressed on cells may bind CD47 ligands, the ligand engagement does not induce CD47 signaling to cause apoptosis. We found that ligation of hSIRPα-Fc proteins with autogenous, but not cross-dressed, CD47 on Jurkat cells induces cell death. This study suggests that using a pig vascularized organ with hCD47 overexpression in some cells, which are more sensitive to macrophage attack but relatively resistant to CD47 signaling-induced deleterious effects, may improve the outcomes of xenotransplantation.

Emerging evidence indicates that CD47-SIRPα signaling plays an important role in regulating DC activation, and hence, T cell priming. The initial evidence for a role of CD47 in controlling DC activation was obtained in a mouse model of DST, in which DST using WT cells induces donor-specific tolerance, but DST using CD47-deficient cells paradoxically induces SIRPα^+^ DC activation and augments anti-donor T cell responses (Wang et al., 2010). A similar finding was made in a mouse model of hepatocyte allotransplantation where WT hepatocytes promote allograft survival, but CD47KO hepatocytes exacerbated rejection (Zhang et al., 2016). Although it was not tested directly, it is conceivable that CD47 cross-dressed on cells, which induces sufficient SIRPα signaling to inhibit macrophages, may suppress SIRPα^+^ DC activation. Thus, in addition to inhibition of phagocytosis, CD47 cross-dressing may also attenuate anti-donor T cell responses when transplants are performed using CD47-overexpressing donors.

CD47-SIRPα signaling is an important component of the tumor immune microenvironment. CD47 upregulation was found in many types of cancer cells, in which CD47 provides an important mechanism to evade macrophage killing (Jaiswal et al., 2009; Chan et al., 2009). CD47 upregulation in tumors also significantly contributes to tumor-induced T cell suppression, as the antitumor activity of CD47 blocking treatment is associated with CD11c^+^ DC activation and is largely T cell-dependent (Liu et al., 2015b, Chen et al., 2020; Li et al., 2020). In the present study, CD47 cross-dressing was found in human T-cell leukemia Jurkat and pig B-lymphoma LCL cells, suggesting a possible involvement of CD47 cross-dressing in the formation of a tumor immunosuppressive microenvironment. In addition, CD47 on EVs and CD47 cross-dressed on tumor cells may also neutralize CD47 ligands, such as TSP-1 that has been shown to induce CD47 activation leading to apoptosis in tumor and endothelial cells (Martinez-Torres et al., 2015), hence, favoring tumor growth.

While the mechanisms of EV-mediated exchange of biological information and materials between cells remain poorly understood (Raposo and Stahl, 2019), EV-induced antigen cross-dressing has been reported to play an important role in the regulation of immune responses, including alloantigen recognition and allograft rejection (Zeng and Morelli, 2018; Gonzalez-Nolasco et al., 2018). Earlier studies have shown that CD47, as a ‘don’t eat me’ signal, is essential for EVs to elicit biological function by preventing their clearance by macrophages (Kamerkar et al., 2017). Here we report that EV-induced CD47 cross-dressing possesses partial activity of the autogenous CD47, such as the ability to initiate inhibitory SIRPα signaling, and therefore offers means of separating the desired and harmful effects of CD47.

## Materials and methods

### Cell culture

Jurkat (J.RT3-T3.5) cell line was purchased from ATCC (named WT Jurkat to distinguish from CD47KO Jurkat cells) and the identity has been authenticated by STR profiling provided by China Center for Type Culture Collection (CCTCC). Jurkat cells were grown in Dulbecco's Modified Eagle's Medium supplemented with 10% Fetal Bovine Serum (FBS) (Atlanta Biologicals), GlutaMax and 100 U/ml penicillin and streptomycin. Pig LCL cell is a pig B cell lymphoma cell line (LCL-13271) derived from Swine Leukocyte Antigen AD (SLA^AD^) miniature swine with post-transplantation lymphoproliferative disease (PTLD), which is kindly provided by Christene Huang (Harvard Medical School). The markers used for characterization of pig LCL cells are CD2, CD25 and anti-mu heavy chain (details were published previously) (Cho et al., 2007). Human CD47 (hCD47) transgenic (tg) pig B-LCL cell line (hCD47-tg LCL) and control pig LCL cell line were generated by transfecting LCL cells with pKS336-hCD47 or empty pKS336 vector, respectively, as described previously (Ide et al., 2007). Pig LCL cells were grown in RPMI 1640 Medium supplemented with 10% FBS, GlutaMax, MEM Non-Essential Amino Acids Solution, sodium pyruvate, 5 μM 2-mercaptoenthaol and 100 U/ml penicillin and streptomycin. Human CD47-tg porcine BMCs were harvested from SLA-defined miniature swine with hCD47 transgene (Watanabe et al., 2020), and BMCs were grown in Dulbecco's Modified Eagle's Medium supplemented with 10% FBS, GlutaMax and 100 U/ml penicillin and streptomycin. Pig aortic endothelial cells (PAOC) immortalized with SV40 were purchased from ABM (catalog # T0448), and the endothelial cell characterization was determined by expression of VE cadherin and CD31, and uptake of fluorescent Dil-Ac-LDL (measured by DiI-Ac-LDL Kit; Cell Applications, Cat # 022K). All PAOC cell lines were grown in Porcine Endothelial Cell Growth Medium (Cell Applications, Cat # P211-500) supplemented with 10% FBS. All cell lines were confirmed negative for mycoplasma contamination by MycoStrip mycoplasma detection kit (InvivoGen, Cat# rep-mys-10). Trypsin/EDTA was used for cell dissociation. Media and reagents for cell culture were purchased from GIBCO. Where indicated, cells were treated with mitomycin C (2 µg/mL for 30 min; Sigma-Aldrich) or cytochalasin D (2 µg/mL for 60 min; Thermo Fisher).

### Generation of CD47KO Jurkat and PAOC sublines

CRISPR small guide RNA (sgRNA) for disrupting hCD47 in Jurkat cells was designed using the online tools (https://crispr.mit.edu), with sequence targeting the exon 2 of hCD47 (CTACTGAAGTATACGTAAAG-TGG [PAM]). The sgRNA was cloned into the pL-CRISPR.EFS.GFP lentiviral vector which was a gift from Benjamin Ebert (Addgene plasmid no. 57818) for co-expression with Cas9 (Heckl et al., 2014). Lentiviral particles were produced by co-transfection of a three-plasmid system consisting of the pL-CRISPR.EFS. GFP vector and packaging plasmids (pVSV-G and pΔ) using CaCl_2_ into 293T cells in 175 cm^2^ flasks. Lentivirus supernatant was collected 48 hr post-transfection, concentrated by ultracentrifugation at 22,000 rpm for 2.5 hr (Beckman Coulter, Optima XE-90) and stored at –80°C until use. GFP^+^ cells were sorted 3 days after lentivirus transduction, then sorted GFP^+^ cells were expanded and assessed for CD47 expression by staining with BV786-conjugated anti-hCD47 mAb B6H12 (BD Bioscience) and PE-conjugated anti-hCD47 mAb CC2C6 (Biolegend). CD47-negative Jurkat cells were established by four rounds of cell sorting. We also generated Jurkat cells that express only transgenic hCD47 by transduction of CD47-negative Jurkat cells with pLVX lentiviral vector encoding hCD47 isoform 2 cDNA.

PAOC cells were immortalized with Lenti-hTERT virus (ABM; cat no. G200) following manufacturer’s instructions and clonal sorting/expansion. Alpha GAL KO pAOC-SV40-hTERT (GTKO) cell line (PAOC/CD47^p^) was created via nucleofection of pAOC-SV40-hTERT with plasmid expressing Cas9 protein (GeneART CRISPR Nuclease Vector, Invitrogen) and guide RNA targeting GGTA-1 gene (aGal protein, guide RNA sequence used: TCATGGTGGATGATATCTCC) using Lonza 4D-Nucleofector, followed by clonal sorting/expansion. PAOC/CD47^null^ was created via nucleofection of PAOC/CD47^p^ cells with plasmid expressing Cas9 protein and guide RNA targeting the pig CD47 gene (guide RNA sequence used: TCACCATCAGAATTACTACA) using Lonza 4D-Nucleofector. PAOC/CD47^null^ and PAOC/CD47^p^ cell lines expressing hCD47 short (305aa, 16aa short intracellular domain, NM_198793, NP_942088, PAOC/CD47^h2^, and PAOC/CD47^p/h2^) or hCD47long isoforms (323aa, 34aa long intracellular domain, NM_001777, NP_001768, PAOC/CD47^h4^, and PAOC/CD47^p/h4^) (Reinhold et al., 1995) were created via nucleofection with plasmid expressing Cas9 protein (GeneART CRISPR Nuclease Vector, Invitrogen) and guide RNA targeting AAVS1 safe harbor site using Lonza 4D-Nucleofector, followed by clonal sorting/expansion.

### Flow cytometric analysis

CD47 expression or cross-dressing on cells was determined by direct staining with BV786- or AF647-conjugated anti human specific CD47 mAb B6H12 (abbreviated anti-hCD47; BD Bioscience) or PE-conjugated anti-human CD47 mAb CC2C6 (with cross-reactivity to pig CD47 and thus, referred to as anti-h/pCD47; Biolegend). The level of cell surface CD47 is expressed as median fluorescent intensity. FITC(fluorescein isothiocyanate)-conjugated anti-pig SIRPα mAb (clone BL1H7) was from Abcam; recombinant human SIRPα/CD172a Fc chimera protein, CF (cat no. 4546-SA-050) was from R&D system; APC-conjugated anti-human IgG Fc (clone HP6017), PE-conjugated anti-human CD90 (clone 5E10), AF647-conjugated anti-human HLA-ABC (clone W6/32) were from Biolegend; PKH26 and PKH67 were from Sigma-Aldrich. For analysis of CD47 expression on human PBMCs, single-cell suspensions were incubated with anti-hCD47 mAb B6H12 in combination with fluorochrome-conjugated anti-human CD45 (clone HI30), CD19 (clone HIB19), CD3 (clone SK7), and CD14 (clone M5E2; all from Biolegend). Dead cells were identified by staining with propidium iodide or 7-AAD. All samples were collected on Flow Cytometer (Fortessa and Celesta, Becton Dickinson), and data were analyzed by Flowjo software (Tree Star).

### Purification of extracellular vesicles

EVs and Exos from cell culture supernatants were purified by a standard differential centrifugation protocol as previously reported (Chen et al., 2018). In brief, bovine Exos were depleted from FBS by overnight centrifugation at 100,000 g, PAOC/CD47^h2^ cells were cultured in media supplemented with 10% Exos-depleted FBS for EVs and Exos purification from cell culture supernatants. Supernatants collected from 48-hr cell cultures were centrifuged at 2000 g (3000 rpm) for 20 min to remove cell debris and dead cells. EVs were pelleted after centrifugation at 16,500 g (9800 rpm) for 45 min (Beckman Coulter, Optima XE-90) and resuspended in PBS. The pelleted Exos from above supernatants were further centrifuged at 100,000 g (26,450 rpm) for 2 hr at 4°C (Beckman Coulter, Optima XE-90) and resuspended in PBS. EVs and Exos from total 1.3×10^7^ PAOC/CD47^h2^ cells cultured for 48 hr were concentrated in 250 ul and 400 ul PBS, respectively, 10 ul of each was used for CD47 cross-dressing.

### Preparation of human macrophages

Blood from healthy volunteers was used to prepare peripheral blood mononuclear cells (PBMCs) by density gradient centrifugation. PBMCs were added at 3×10^6^ per well in a 24-well plate, and unattached cells were removed from the plate on the second day. Attached cells were then differentiated to macrophages by 8–9 days of culture in Iscove's Modified Dulbecco's Medium (IMDM) (Gibco) + GlutaMax (Thermo fisher scientific) supplemented with 10% AB human serum (Gemini Bio-products, Inc), containing 10 ng/ml human M-CSF (PeproTech) and 100 U/ml penicillin and streptomycin (Gibco). The use of human blood samples was approved by the Institutional Review Board of Columbia University Medical Center.

### Flow cytometry-based phagocytic assay

Macrophages generated as above were harvested from plates using Trypsin-EDTA (Thermo fisher scientific). The indicated target cells were labeled with Celltrace violet (Thermo fisher scientific) according to the manufacturer’s protocol, and phagocytic assay was performed by co-culturing 6×10^4^ Celltrace Violet-labeled target cells with 3×10^4^ human macrophages for 2 hr in ultra-low attachment 96-well flat bottom plates in IMDM + GlutaMax without antibiotics or serum added. All cells were harvested after coculture, and phagocytosis was determined by flow cytometry analyses, in which the phagocytic ratio is calculated as the percentage of macrophages that engulfed target cells (human CD45^+^CD14^+^Celltrace violet^+^) among total macrophages (human CD45^+^CD14^+^).

### Statistical analysis

Data were analyzed using GraphPad Prism (version 8; San Diego, CA) and presented as mean value ± SDs. The level of significant differences in group means was assessed by student’s t-test, and a p-value of ≤0.05 was considered significant in all analyses herein.

## Data Availability

Figure 1, 4, 5; Figure 2-figure supplement 2, Figure 2-figure supplement 3, Figure 3-figure supplement 1, Figure 3-figure supplement 2, Figure 5-figure supplement 1, Figure 5-figure supplement 3 - Source Data contain the numerical data used to generate the figures.

## Additional files

MDAR checklist

Source data 1. CD47 crossdressing source data E-life R1.

## References

[bib1] Abe M, Cheng J, Qi J, Glaser RM, Thall AD, Sykes M, Yang YG (2002). Elimination of porcine hemopoietic cells by macrophages in mice. Journal of Immunology.

[bib2] Boettcher AN, Cunnick JE, Powell EJ, Egner TK, Charley SE, Loving CL, Tuggle CK (2019). Porcine signal regulatory protein alpha binds to human CD47 to inhibit phagocytosis: implications for human hematopoietic stem cell transplantation into severe combined immunodeficient pigs. Xenotransplantation.

[bib3] Chan KS, Espinosa I, Chao M, Wong D, Ailles L, Diehn M, Gill H, Presti J, Chang HY, van de Rijn M, Shortliffe L, Weissman IL (2009). Identification, molecular characterization, clinical prognosis, and therapeutic targeting of human bladder tumor-initiating cells. PNAS.

[bib4] Chao MP, Alizadeh AA, Tang C, Myklebust JH, Varghese B, Gill S, Jan M, Cha AC, Chan CK, Tan BT, Park CY, Zhao F, Kohrt HE, Malumbres R, Briones J, Gascoyne RD, Lossos IS, Levy R, Weissman IL, Majeti R (2010). Anti-CD47 antibody synergizes with rituximab to promote phagocytosis and eradicate non-Hodgkin lymphoma. Cell.

[bib5] Chen G, Huang AC, Zhang W, Zhang G, Wu M, Xu W, Yu Z, Yang J, Wang B, Sun H, Xia H, Man Q, Zhong W, Antelo LF, Wu B, Xiong X, Liu X, Guan L, Li T, Liu S, Yang R, Lu Y, Dong L, McGettigan S, Somasundaram R, Radhakrishnan R, Mills G, Lu Y, Kim J, Chen YH, Dong H, Zhao Y, Karakousis GC, Mitchell TC, Schuchter LM, Herlyn M, Wherry EJ, Xu X, Guo W (2018). Exosomal PD-L1 contributes to immunosuppression and is associated with anti-PD-1 response. Nature.

[bib6] Chen M, Wang Y, Wang H, Sun L, Fu Y, Yang Y-G (2019). Elimination of donor CD47 protects against vascularized allograft rejection in mice. Xenotransplantation.

[bib7] Chen H, Cong X, Wu C, Wu X, Wang J, Mao K, Li J, Zhu G, Liu F, Meng X, Song J, Sun X, Wang X, Liu S, Zhang S, Yang X, Song Y, Yang Y-G, Sun T (2020). Intratumoral delivery of CCL25 enhances immunotherapy against triple-negative breast cancer by recruiting CCR9+ T cells. Science Advances.

[bib8] Cho PS, Lo DP, Wikiel KJ, Rowland HC, Coburn RC, McMorrow IM, Goodrich JG, Arn JS, Billiter RA, Houser SL, Shimizu A, Yang YG, Sachs DH, Huang CA (2007). Establishment of transplantable porcine tumor cell lines derived from MHC-inbred miniature swine. Blood.

[bib9] Cooper JA (1987). Effects of cytochalasin and phalloidin on actin. The Journal of Cell Biology.

[bib10] Deuse T, Hu X, Gravina A, Wang D, Tediashvili G, De C, Thayer WO, Wahl A, Garcia JV, Reichenspurner H, Davis MM, Lanier LL, Schrepfer S (2019). Hypoimmunogenic derivatives of induced pluripotent stem cells evade immune rejection in fully immunocompetent allogeneic recipients. Nature Biotechnology.

[bib11] Gao Q, Chen K, Gao L, Zheng Y, Yang Y-G (2016). Thrombospondin-1 signaling through CD47 inhibits cell cycle progression and induces senescence in endothelial cells. Cell Death & Disease.

[bib12] Gao L, Chen K, Gao Q, Wang X, Sun J, Yang Y-G (2017). Cd47 deficiency in tumor stroma promotes tumor progression by enhancing angiogenesis. Oncotarget.

[bib13] Gonzalez-Nolasco B, Wang M, Prunevieille A, Benichou G (2018). Emerging role of exosomes in allorecognition and allograft rejection. Current Opinion in Organ Transplantation.

[bib14] Guilliams M, Dutertre C-A, Scott CL, McGovern N, Sichien D, Chakarov S, Van Gassen S, Chen J, Poidinger M, De Prijck S, Tavernier SJ, Low I, Irac SE, Mattar CN, Sumatoh HR, Low GHL, Chung TJK, Chan DKH, Tan KK, Hon TLK, Fossum E, Bogen B, Choolani M, Chan JKY, Larbi A, Luche H, Henri S, Saeys Y, Newell EW, Lambrecht BN, Malissen B, Ginhoux F (2016). Unsupervised high-dimensional analysis aligns dendritic cells across tissues and species. Immunity.

[bib15] Han X, Wang M, Duan S, Franco PJ, Kenty JHR, Hedrick P, Xia Y, Allen A, Ferreira LMR, Strominger JL, Melton DA, Meissner TB, Cowan CA (2019). Generation of hypoimmunogenic human pluripotent stem cells. PNAS.

[bib16] Heckl D, Kowalczyk MS, Yudovich D, Belizaire R, Puram RV, McConkey ME, Thielke A, Aster JC, Regev A, Ebert BL (2014). Generation of mouse models of myeloid malignancy with combinatorial genetic lesions using CRISPR-Cas9 genome editing. Nature Biotechnology.

[bib17] Ide K, Wang H, Tahara H, Liu J, Wang X, Asahara T, Sykes M, Yang YG, Ohdan H (2007). Role for CD47-sirpalpha signaling in xenograft rejection by macrophages. PNAS.

[bib18] Isenberg JS, Roberts DD (2019). The role of CD47 in pathogenesis and treatment of renal ischemia reperfusion injury. Pediatric Nephrology.

[bib19] Jaiswal S, Jamieson CHM, Pang WW, Park CY, Chao MP, Majeti R, Traver D, van Rooijen N, Weissman IL (2009). Cd47 is upregulated on circulating hematopoietic stem cells and leukemia cells to avoid phagocytosis. Cell.

[bib20] Kamerkar S, LeBleu VS, Sugimoto H, Yang S, Ruivo CF, Melo SA, Lee JJ, Kalluri R (2017). Exosomes facilitate therapeutic targeting of oncogenic KRAS in pancreatic cancer. Nature.

[bib21] Li Y, Zhang M, Wang X, Liu W, Wang H, Yang Y-G (2020). Vaccination with CD47 deficient tumor cells elicits an antitumor immune response in mice. Nature Communications.

[bib22] Liu X, Pu Y, Cron K, Deng L, Kline J, Frazier WA, Xu H, Peng H, Fu YX, Xu MM (2015a). Cd47 blockade triggers T cell-mediated destruction of immunogenic tumors. Nature Medicine.

[bib23] Liu J, Wang L, Zhao F, Tseng S, Narayanan C, Shura L, Willingham S, Howard M, Prohaska S, Volkmer J, Chao M, Weissman IL, Majeti R (2015b). Pre-clinical development of a humanized anti-CD47 antibody with anti-cancer therapeutic potential. PLOS ONE.

[bib24] Martinez-Torres AC, Quiney C, Attout T, Boullet H, Herbi L, Vela L, Barbier S, Chateau D, Chapiro E, Nguyen-Khac F, Davi F, Le Garff-Tavernier M, Moumné R, Sarfati M, Karoyan P, Merle-Béral H, Launay P, Susin SA (2015). Cd47 agonist peptides induce programmed cell death in refractory chronic lymphocytic leukemia B cells via PLCγ1 activation: evidence from mice and humans. PLOS Medicine.

[bib25] Mateo V, Lagneaux L, Bron D, Biron G, Armant M, Delespesse G, Sarfati M (1999). Cd47 ligation induces caspase-independent cell death in chronic lymphocytic leukemia. Nature Medicine.

[bib26] Meijles DN, Sahoo S, Al Ghouleh I, Amaral JH, Bienes-Martinez R, Knupp HE, Attaran S, Sembrat JC, Nouraie SM, Rojas MM, Novelli EM, Gladwin MT, Isenberg JS, Cifuentes-Pagano E, Pagano PJ (2017). The matricellular protein TSP1 promotes human and mouse endothelial cell senescence through CD47 and Nox1. Science Signaling.

[bib27] Navarro-Alvarez N, Yang Y-G (2014). Lack of CD47 on donor hepatocytes promotes innate immune cell activation and graft loss: a potential barrier to hepatocyte xenotransplantation. Cell Transplantation.

[bib28] Nomura S, Ariyoshi Y, Watanabe H, Pomposelli T, Takeuchi K, Garcia G, Tasaki M, Ayares D, Sykes M, Sachs D, Johnson R, Yamada K (2020). Transgenic expression of human CD47 reduces phagocytosis of porcine endothelial cells and podocytes by baboon and human macrophages. Xenotransplantation.

[bib29] Oldenborg PA, Zheleznyak A, Fang YF, Lagenaur CF, Gresham HD, Lindberg FP (2000). Role of CD47 as a marker of self on red blood cells. Science.

[bib30] Raposo G, Stahl PD (2019). Extracellular vesicles: a new communication paradigm?. Nature Reviews. Molecular Cell Biology.

[bib31] Reinhold MI, Lindberg FP, Plas D, Reynolds S, Peters MG, Brown EJ (1995). In vivo expression of alternatively spliced forms of integrin-associated protein (CD47). Journal of Cell Science.

[bib32] Saumet A, Slimane MB, Lanotte M, Lawler J, Dubernard V (2005). Type 3 repeat/C-terminal domain of thrombospondin-1 triggers caspase-independent cell death through CD47/alphavbeta3 in promyelocytic leukemia NB4 cells. Blood.

[bib33] Sosale NG, Ivanovska II, Tsai RK, Swift J, Hsu JW, Alvey CM, Zoltick PW, Discher DE (2016). “ Marker of self” CD47 on lentiviral vectors decreases macrophage-mediated clearance and increases delivery to SIRPA-expressing lung carcinoma tumors. Molecular Therapy. Methods & Clinical Development.

[bib34] Takeuchi K, Ariyoshi Y, Shimizu A, Okumura Y, Cara-Fuentes G, Garcia GE, Pomposelli T, Watanabe H, Boyd L, Ekanayake-Alper DK, Amarnath D, Sykes M, Sachs DH, Johnson RJ, Yamada K (2021). Expression of human CD47 in pig glomeruli prevents proteinuria and prolongs graft survival following pig-to-baboon xenotransplantation. Xenotransplantation.

[bib35] Tena AA, Sachs DH, Mallard C, Yang Y-G, Tasaki M, Farkash E, Rosales IA, Colvin RB, Leonard DA, Hawley RJ (2017). Prolonged survival of pig skin on baboons after administration of pig cells expressing human CD47. Transplantation.

[bib36] Wang H, Madariaga ML, Wang S, Van Rooijen N, Oldenborg PA, Yang YG (2007a). Lack of CD47 on nonhematopoietic cells induces split macrophage tolerance to cd47null cells. PNAS.

[bib37] Wang H, VerHalen J, Madariaga ML, Xiang S, Wang S, Lan P, Oldenborg PA, Sykes M, Yang YG (2007b). Attenuation of phagocytosis of xenogeneic cells by manipulating CD47. Blood.

[bib38] Wang H, Wu X, Wang Y, Oldenborg PA, Yang YG (2010). Cd47 is required for suppression of allograft rejection by donor-specific transfusion. Journal of Immunology.

[bib39] Wang C, Wang H, Ide K, Wang Y, Van Rooijen N, Ohdan H, Yang Y-G (2011). Human CD47 expression permits survival of porcine cells in immunodeficient mice that express SIRPα capable of binding to human CD47. Cell Transplantation.

[bib40] Wang Y, Wang H, Bronson R, Fu Y, Yang Y-G (2014). Rapid dendritic cell activation and resistance to allotolerance induction in anti-CD154-treated mice receiving CD47-deficient donor-specific transfusion. Cell Transplantation.

[bib41] Watanabe H, Ariyoshi Y, Pomposelli T, Takeuchi K, Ekanayake-Alper DK, Boyd LK, Arn SJ, Sahara H, Shimizu A, Ayares D, Lorber MI, Sykes M, Sachs DH, Yamada K (2020). Intra-bone bone marrow transplantation from hcd47 transgenic pigs to baboons prolongs chimerism to > 60 days and promotes increased porcine lung transplant survival. Xenotransplantation.

[bib42] Weiskopf K, Jahchan NS, Schnorr PJ, Cristea S, Ring AM, Maute RL, Volkmer AK, Volkmer J-P, Liu J, Lim JS, Yang D, Seitz G, Nguyen T, Wu D, Jude K, Guerston H, Barkal A, Trapani F, George J, Poirier JT, Gardner EE, Miles LA, de Stanchina E, Lofgren SM, Vogel H, Winslow MM, Dive C, Thomas RK, Rudin CM, van de Rijn M, Majeti R, Garcia KC, Weissman IL, Sage J (2016). CD47-blocking immunotherapies stimulate macrophage-mediated destruction of small-cell lung cancer. The Journal of Clinical Investigation.

[bib43] Willingham SB, Volkmer JP, Gentles AJ, Sahoo D, Dalerba P, Mitra SS, Wang J, Contreras-Trujillo H, Martin R, Cohen JD, Lovelace P, Scheeren FA, Chao MP, Weiskopf K, Tang C, Volkmer AK, Naik TJ, Storm TA, Mosley AR, Edris B, Schmid SM, Sun CK, Chua MS, Murillo O, Rajendran P, Cha AC, Chin RK, Kim D, Adorno M, Raveh T, Tseng D, Jaiswal S, Enger PØ, Steinberg GK, Li G, So SK, Majeti R, Harsh GR, van de Rijn M, Teng NNH, Sunwoo JB, Alizadeh AA, Clarke MF, Weissman IL (2012). The CD47-signal regulatory protein alpha (sirpa) interaction is a therapeutic target for human solid tumors. PNAS.

[bib44] Yang Y-G, Sykes M (2007). Xenotransplantation: current status and a perspective on the future. Nature Reviews. Immunology.

[bib45] Yang Y-G (2010). Cd47 in xenograft rejection and tolerance induction. Xenotransplantation.

[bib46] Zeng F, Morelli AE (2018). Extracellular vesicle-mediated MHC cross-dressing in immune homeostasis, transplantation, infectious diseases, and cancer. Seminars in Immunopathology.

[bib47] Zhang M, Wang H, Tan S, Navarro-Alvarez N, Zheng Y, Yang Y-G (2016). Donor CD47 controls T cell alloresponses and is required for tolerance induction following hepatocyte allotransplantation. Scientific Reports.

